# Soundscape Characterization Using Autoencoders and Unsupervised Learning

**DOI:** 10.3390/s24082597

**Published:** 2024-04-18

**Authors:** Daniel Alexis Nieto-Mora, Maria Cristina Ferreira de Oliveira, Camilo Sanchez-Giraldo, Leonardo Duque-Muñoz, Claudia Isaza-Narváez, Juan David Martínez-Vargas

**Affiliations:** 1 Máquinas Inteligentes y Reconocimiento de Patrones (MIRP), Instituto Tecnológico Metropolitano ITM, Medellín 050034, Colombia; leonardoduque@itm.edu.co; 2 Instituto de Ciências Matemáticas e de Computação, Universidade de São Paulo, São Carlos 13566-590, SP, Brazil; cristina@icmc.usp.br; 3 Grupo Herpetológico de Antioquia, Institute of Biology, Universidad de Antioquia-UdeA, Medellín 050010, Colombia; camilo.sanchez@udea.edu.co; 4 SISTEMIC, Facultad de Ingeniería, Universidad de Antioquia-UdeA, Medellín 050010, Colombia; victoria.isaza@udea.edu.co; 5 GIDITIC, Universidad EAFIT, Medellín 050022, Colombia

**Keywords:** autoencoders, deep learning, ecoacoustics, landscape monitoring, soundscape ecology, unsupervised learning

## Abstract

Passive acoustic monitoring (PAM) through acoustic recorder units (ARUs) shows promise in detecting early landscape changes linked to functional and structural patterns, including species richness, acoustic diversity, community interactions, and human-induced threats. However, current approaches primarily rely on supervised methods, which require prior knowledge of collected datasets. This reliance poses challenges due to the large volumes of ARU data. In this work, we propose a non-supervised framework using autoencoders to extract soundscape features. We applied this framework to a dataset from Colombian landscapes captured by 31 audiomoth recorders. Our method generates clusters based on autoencoder features and represents cluster information with prototype spectrograms using centroid features and the decoder part of the neural network. Our analysis provides valuable insights into the distribution and temporal patterns of various sound compositions within the study area. By utilizing autoencoders, we identify significant soundscape patterns characterized by recurring and intense sound types across multiple frequency ranges. This comprehensive understanding of the study area’s soundscape allows us to pinpoint crucial sound sources and gain deeper insights into its acoustic environment. Our results encourage further exploration of unsupervised algorithms in soundscape analysis as a promising alternative path for understanding and monitoring environmental changes.

## 1. Introduction

Acoustic landscape ecology, also known as soundscape ecology, is a field within ecoacoustics dedicated to describing and studying the sounds present in natural landscapes [1]. The goal is to extract information about various types of sounds or sources originating from human activity (anthropophonies), physical phenomena (geophonies), and biotic sources (biophonies), the latter referring to sounds emitted by living organisms such as animal vocalizations [1,2,3]. These acoustic signals serve as valuable indicators for assessing species diversity and abundance, habitat use, and population dynamics [4]. Additionally, changes in the acoustic landscape, often influenced by human activities, can reflect the impacts of habitat loss and degradation on wildlife populations [5]. In this context, the soundscape emerges as a crucial area for monitoring the ecological integrity of landscapes and detecting early signals of ecological change. Studying soundscapes can, thus, significantly contribute to biodiversity conservation efforts.

Soundscape studies employ acoustic recording units (ARUs) to capture sound over predefined periods [6], which can range from days to weeks or even months. ARUs are programmed to be activated at specified intervals, recording audio for a predetermined duration before entering a standby mode for a set period and then resuming recording. Recent advancements in recording technology, characterized by low energy consumption and increased storage capacity, have facilitated operation over extended sampling periods and recording times. Consequently, significant volumes of soundscape data are being generated, creating a demand for the development of automated tools for efficient data processing and analysis [7].

Machine learning is now a popular solution for developing innovative data processing frameworks due to its exceptional performance across various domains, including computer vision, semantic analysis, natural language processing, automatic speech, audio recognition, and machinery fault prevention and diagnosis [8,9]. Within the realm of ecoacoustics and soundscape analysis, there is extensive usage of machine learning algorithms such as Random Forests [10], Support Vector Machines [11], and Neural Networks [1,12] to extract meaningful features from acoustic data. Recent advancements in ecoacoustics and soundscape research have leveraged deep features and Neural Networks to enhance sound type identification. For instance, Dufourq et al. [13] employed transfer learning to adapt existing Convolutional Neural Networks (CNNs) for bioacoustic classification. Furthermore, architectures such as ResNet, EfficientNet, MobileNet, and DesNet have been deployed to accurately identify acoustic scenes involving humans, birds, insects, and silence [14], and the MobileNetV2 architecture has been successfully employed for classifying biophonies, geophonies, anthropophonies, and silence [1]. These advancements underscore the growing contribution of machine learning to advance our understanding of acoustic environments.

Supervised methodologies in acoustic analysis often yield high-accuracy results but face challenges due to their reliance on labeled data for model training and testing. These challenges include the need for expert analysis to identify patterns in audio [15,16], assumptions about data structure [10] such as the set of possible animal vocalizations [17], time-consuming sample labeling with practical issues like lacking specific timestamps and frequency bands, and handling overlapping acoustic events in time and frequency [15,16]. Moreover, feature extraction methods are sensitive to noisy data [7]. These limitations drive the exploration of unsupervised learning as a compelling alternative. Unsupervised methods can circumvent many difficulties inherent in supervised approaches. For instance, Keen et al. proposed a framework combining unsupervised Random Forest, K-means clustering, and t-SNE to evaluate acoustic diversity [10]. Ulloa et al. introduced the Multiresolution Analysis of Acoustic Diversity (MAAD) method, which decomposes the acoustic community into elementary components called soundtypes based on their time and frequency attributes [18]. These contributions pave promising paths for leveraging unsupervised learning to understand and explore soundscape composition beyond specific sound types, often limited to biophonies, thus capturing valuable information across acoustic environments.

In this paper, we introduce a novel methodology for characterizing soundscapes using autoencoders, providing a fresh perspective that goes beyond traditional species-specific approaches. Our approach utilizes autoencoders to effectively uncover large-scale patterns within sound recordings, thereby capturing the broader ecological context. By focusing on feature extraction with autoencoders, we aim to bridge the gap between identified patterns, metadata, and the ecological attributes of the landscape. This integration enables a comprehensive evaluation of the soundscape, considering both acoustic patterns and their ecological significance. Moreover, we introduce an unsupervised framework designed to explore novel landscape attributes through the lens of soundscape heterogeneity. This framework promotes a holistic understanding of the acoustic environment, facilitating the identification and interpretation of previously unexplored patterns and associations. In summary, the novelty of our work lies in the synthesis of cutting-edge autoencoder technology with soundscape ecology, offering a forward-thinking approach to characterize and interpret acoustic landscapes within a broader ecological context.

## 2. Related Work

The majority of deep learning methodologies for ecoacoustics applications reported in the literature rely on supervised approaches, whereas end-to-end unsupervised frameworks have not been sufficiently studied. However, some approximations have emerged, e.g., Rowe et al. [19] used autoencoders to characterize sound types and identify groups corresponding to species vocalizations. Nevertheless, although the methodology is unsupervised, it requires prior information about the species for validation. Dias et al. [20] also explored autoencoders for feature extraction and data visualization; additional, they used acoustic indices and spectral features to characterize sites from Costa Rica and Brazil. Best et al. [21] introduced a new method for encoding vocalizations, using an autoencoder network to obtain embeddings from eight datasets across six species, including birds and marine mammals, also employing clustering and dimension reduction techniques such as DBSCAN and UMAP. Akbal et al. [22] collected a new anuran sound dataset and proposed a hand-modeled sound classification system through an improved one-dimensional local binary pattern (1D-LBP) and Tunable Q Wavelet Transform (TQWT), obtaining a 99.35% accuracy in classifying 26 anuran species. Gibb et al. [15] discuss the limitations of supervision in soundscape analysis and propose variational autoencoders to embed latent features from acoustic survey data and evaluate habitat degradation. Rendon et al. [23] proposed the Uncertainty Fréchet Clustering Internal Validity Index, which was assessed using real-world and synthetic data, including a soundscape dataset identifying the transformation of ecosystems. On the other hand, Allaoi et al. [24] investigated the problem of treating embedding and clustering simultaneously to uncover data structure reliably by constraining manifold embedding through clustering and introduce the UEC method. Given the evolution and significant interest within the scientific community regarding this study area, we conducted a comprehensive review of machine learning applications in soundscape ecology and ecoacoustics. In this review, we compiled a list of methods encompassing both supervised and unsupervised learning, as well as deep and traditional approaches [25]. Similarly, we summarize the latest highly related works in Table 1.

This review of related work highlights the importance of further investigating unsupervised methodologies to facilitate the exploration of acoustic data and assist with the identification of representative patterns associated with landscape heterogeneity. Moreover, one observes a trend towards investigating and relating heterogeneity with landscape attributes indicators of ecosystem health.

## 3. Materials and Methods

**Figure 1:**
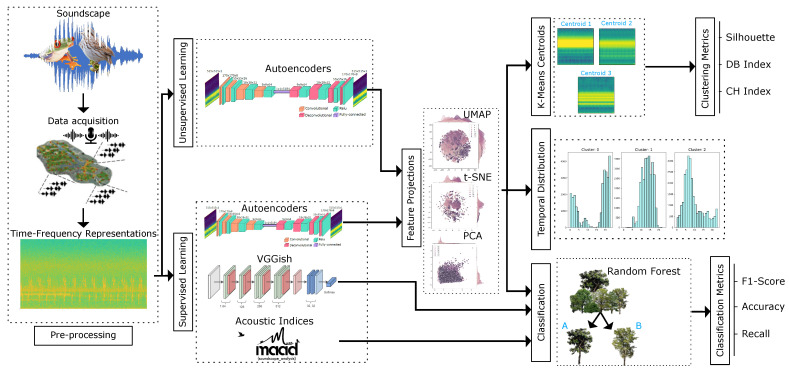
Overview of the principal stages in the methodological framework based on pre-processing, supervised learning, unsupervised learning, clustering analysis and results, and classification results.

The methodology introduced in this work is summarized in Figure 1. The initial step following data acquisition involves generating time-frequency spectrogram representations, which serve as input to train an autoencoder architecture for feature extraction. Two data processing strategies are executed: a supervised pathway and an unsupervised pathway. The supervised data processing pathway gives us a baseline to compare the performance of the autoencoder features against more standard representations adopted in soundscape ecology. We evaluated the performance of a Random Forest classifier on the features learned by the proposed autoencoder, taking, as baselines, features obtained with the VGGish architecture and a feature vector of acoustic indices. Acoustic indices have been proven effective to capture acoustic variations reflecting ecosystem attributes, while the VGGish [26] Deep Neural Network is commonly employed for video and audio analyses. In the unsupervised data processing pathway, features extracted by the autoencoder and their projections are clustered using K-means, and the resulting clusters are characterized based on their temporal patterns and spectral ranges. We utilized multiple metrics of cluster cohesion and separation to establish a quantitative baseline for assessing cluster quality. Further details on this process are provided below.

### 3.1. Study Site

**Figure 2:**
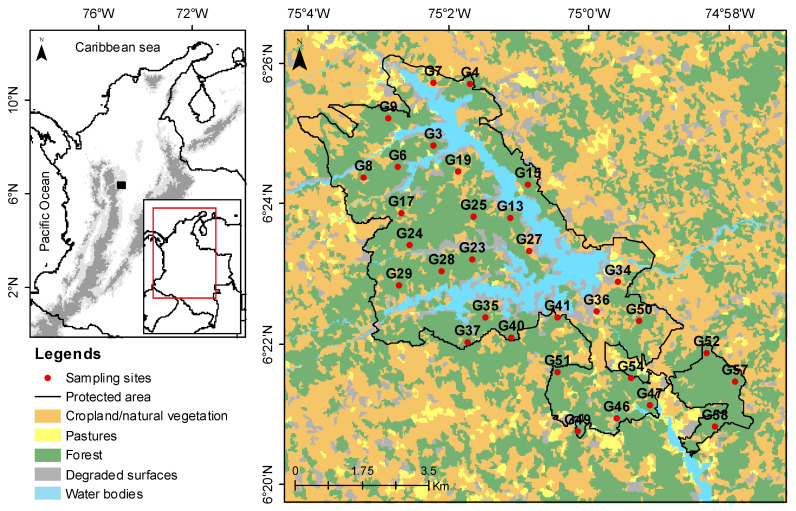
Geographical distribution of recorders (31 Song Meter SM4 devices) in the Jaguas protected area in Antioquia, Colombia.

Our experimental investigation utilized a dataset sourced from the Jaguas Colombian tropical forest. ARUs were placed near the Jaguas hydroelectric power plant in the northern region of Antioquia (6°26′ N, 75°05′ W; 6°21′ N, 74°59′ W) (refer to Figure 2 for the location of the recorders). Jaguas spans a protected area of 50 km², characterized by an elevation gradient from 850 to 1300 m above sea level. The reserve predominantly comprises secondary forests (70%), with the remaining areas consisting of a vegetation mosaic (23%), degraded surfaces (5%), and grassland (2%). Renowned for its rich communities of terrestrial vertebrates, the protected area plays a crucial role in biodiversity conservation at the regional scale [27,28]. For data acquisition, 31 Song Meter SM4 devices (Wildlife Acoustics, Inc., Maynard, MA, USA) were deployed throughout the study site (technical details are summarized in Table 2). Data were sampled for one minute at 44.1 kHz every 15 min, resulting in four .wav files per hour and a total of 20,068 audio files overall. We sub-sampled the dataset to 22,050 because the audible soundtypes of interest occur mostly under 12 KHz. The recorders were configured with 16 dB of gain at a resolution of 16 bits and stereophonic sound. The complete dataset at its original sampling frequency had a size of 212.4 GB. The dataset was collected by the herpetological group of Antioquia (GHA) between 11 May and 26 June 2018.

### 3.2. Pre-Processing

We implemented the method described in [29] for automatically detecting and removing recordings with heavy rainfall. These recordings were excluded due to their high signal-to-noise ratio and the presence of various sound types, such as geophonies (e.g., rivers) and biophonies (e.g., cicadas), which often mask a wide range of frequencies in ecological audio. Removing these instances was necessary to prevent overfitting or biases in the feature extraction and clustering tasks. As a result of this pre-processing step, 16,968 (12,313 forest and 46,151 non-forest) recordings remained for subsequent analysis.

### 3.3. Spectrograms Computation and Parameterization

The data were processed in batches using Python 3.8 and PyTorch 2.2 software. Following standard practice in ecoacoustics data analysis and the knowledge generated around audio featuring using spectral representations [17,30,31,32,33], the audio was converted to the time–frequency domain spectrogram representation using the Short-Time Fourier Transform (STFT), as shown in Equation (1). Each one-minute recording was split into five 12 s segments. This division allowed us to obtain spectrograms with dimensions of 515 × 515 by applying a Hamming window of length 1028 and an overlap equal to half the window size. To maintain a squared output, the number of frequency bins was kept equal to the window length.

(1) $$STFT\left[k,l\right]=\sum_{n=0}^{N-1}s\left(n\right)W\left(n+IL\right)exp\left(-\frac{j2\pi nk}{N}\right).\;0\leq n,k\leq N-1$$

where *n* and *k* are the time and frequency indices, respectively, and *l* is the relative displacement of the current audio segment in terms of steps of *L* samples [34]. The Hamming window is defined as in Equation (2):

(2) $$W\left[n\right]=a_{0}-a_{1}Cos\left(\frac{2\pi n}{N-1}\right).\;a_{0}=0.53836,\;a_{1}=0.46164.$$

An audio segment duration of twelve seconds is unlike the standard practice in the field, which is typically less than five seconds [19]. Our choice of 12 s segments was influenced by the Short-Time Fourier Transform (STFT) parameters, which yield 515 bins in the frequency axis. The effective management of these segments demanded the development of a customized data loader for loading and processing individual segments using the STFT. This process allowed us to generate spectrograms in batches of 14 audio files, yielding 70 sub-audios per batch.

### 3.4. Autoencoders

Autoencoders are Deep Neural Network architectures tailored to leverage the potential of deep learning for automatic and unsupervised feature extraction. The primary objective is to train a multi-layer network capable of learning a low-dimensional embedding from the input high-dimensional data while preserving relevant patterns [35]. Within this architecture, there exists a middle layer where features are extracted via abstract representations derived from convolutional operations across each level of the network [36]. This intermediate layer is commonly referred to as the latent space, and the process of generating this space is known as the encoding phase. Following the encoding phase, a decoding process is carried out using the learned representation within the latent space. The Neural Network is configured to execute the encoding process in an “inverse direction”, meaning features are projected from a low-dimensional representation back to the input space, thereby reconstructing the original data. Theoretically, the outputs of the autoencoder should closely resemble the original data. Consequently, the latent space effectively represents the original input data using fewer dimensions, implying efficient information compression.

Mathematically, let $x\in R^{D}$ be an input vector; an autoencoder maps x to a latent space $z\in R^{D^{\prime }}$ using a deterministic function $f_{\theta_{e}}\left(x\right)=s\left(\mathrm{Wx}+b\right)$, according to the parameters θ={W,b}, where W is a weight matrix and b is a bias. The new representation vector z can be returned to the original input space using a second function $g_{\theta_{d}}\left(z\right)=s\left(W^{\prime }z+b^{\prime }\right)$, with $\theta_{d}=\left\{W^{\prime },b^{\prime }\right\}$ [37,38]. This is represented in Equation (3).

(3) $$\theta_{e}^{*},\theta_{d}^{*}=\arg\min_{\theta_{e},\theta_{d}}\frac{1}{n}\sum_{i=1}^{n}L\left(x^{\left(i\right)},y^{\left(i\right)}\right)$$

Due to *y* resulting from transformations performed with the encoding and decoding functions $f_{\theta },g_{\theta^{\prime }}$, the parameters $\theta_{e}^{*}$ and $\theta_{d}^{*}$ can be expressed in terms of x as in Equation (4):

(4) $$\theta_{e}^{*},\theta_{d}^{*}=\arg\min_{\theta_{e},\theta_{d}}\frac{1}{n}\sum_{i=1}^{n}L\left(x^{\left(i\right)},g_{\theta d}\left(f_{\theta e}\left(x^{\left(i\right)}\right)\right)\right)$$

where *L* denotes the Magnitude Square Error (MSE) loss function. This metric calculates the average Euclidean distance between corresponding pixels of the original and reconstructed spectrograms. The optimization process involves separately adjusting the parameters $\theta_{e}$ and $\theta_{d}$ to minimize the MSE, thereby enhancing the fidelity of the reconstructed spectrograms.

### 3.5. Feature Projection

After processing spectrograms through the autoencoder architecture, the dimension of the latent space is determined by the size of the resulting images and the number of channels. The resulting feature vectors have a size of img_width×img_height×num_channels=9×9×64=5184, which represents a significant reduction in input dimensionality, compared to the original spectrogram dimensions of 515×515 = 265,225. Thus, the original information can be preserved using representations with only 2% of the original image size.

We experimented with PCA, t-SNE, and UMAP for dimension reduction in order to have grounds for a comparative analysis within the context of soundscape data. The techniques have been employed both for visualization purposes and also to reduce the dimensionality of the autoencoder feature space prior to classification.

#### 3.5.1. Principal Component Analysis (PCA)

Principal Component Analysis (PCA) is employed for the dimensionality reduction of datasets characterized by a substantial number of dependent variables. The primary objective is to preserve essential information within the dataset through the transformation of variables into uncorrelated variables known as Principal Components (PCs) [39]. By eliminating redundancy, PCA enhances computational efficiency and reduces the risk of overfitting [40]. We selected the number of principal components maintaining a 90% of the variance.

#### 3.5.2. t-Distributed Stochastic Neighbor Embedding (t-SNE)

t-distributed Stochastic Neighbor Embedding (t-SNE) was introduced as a technique to project high-dimensional data into a low-dimensional representation space [41]. It is a variation of Stochastic Neighbor Embedding (SNE), which compares conditional probabilities representing similarities between data points in different dimensions [41]. Conditional probabilities are calculated from the Euclidean distance between data points $x_{i}$ and $x_{j}$ as in Equation (5).

(5) $$p_{i|j}=\frac{exp\left(-\left|\right|x_{i}-x_{j}{\left|\right|}^{2}/2\sigma_{i}^{2}\right)}{\sum_{k\neq i}exp\left(-\left|\right|x_{i}-x_{k}{\left|\right|}^{2}/2\sigma_{i}^{2}\right)}$$

where $\sigma_{i}$ is the variance of a Gaussian centered on $x_{i}$. Then, the conditional probability $q_{j|i}$ of the projected counterparts $y_{i}$ and $y_{j}$ is obtained as in Equation (6).

(6) $$q_{i|j}=\frac{exp\left(-\left|\right|y_{i}-y_{j}{\left|\right|}^{2}/2\sigma_{i}^{2}\right)}{\sum_{k\neq i}exp\left(-\left|\right|y_{i}-y_{k}{\left|\right|}^{2}/2\sigma_{i}^{2}\right)}$$

The technique seeks to minimize the mismatch between conditional probabilities $p_{i|j}$ and $q_{i|j}$.

#### 3.5.3. Uniform Manifold Approximation and Projection (UMAP)

The UMAP (Uniform Manifold Approximation and Projection) dimension reduction algorithm is grounded in a rigorous mathematical foundation rooted in Riemannian geometry and algebraic topology. It stands as an alternative to t-SNE that notably boasts significantly faster performance compared to most t-SNE implementations, rendering it more efficient for handling large datasets. Moreover, its mathematical formulation seeks the preservation of both local and global structures inherent in the data [42]. Over time, UMAP has emerged as a highly popular non-linear projection technique, particularly for visualizing intricate patterns delineated by features in two or three dimensions [43].

### 3.6. K-Means Clustering

Clustering serves as an unsupervised learning technique for exploratory data analysis, particularly useful when there is limited prior knowledge about the data and its distribution [44]. The underlying principle involves comparing intrinsic features and generating clusters based on similarities or minimum distances computed from the data features. Clustering algorithms are generally categorized into two main groups: probability model-based approaches and non-parametric approaches [45]. The latter is further subdivided into hierarchical or partitional methods, offering a variety of algorithms to choose from [24,45,46,47]. In this study, we employed the K-means algorithm, which falls under the category of partitional algorithms [47,48]. K-means clustering partitions the data into distinct clusters based on the similarity of data points, providing a straightforward and efficient approach to clustering analysis.

Given a dataset $X=x_{1},\ldots ,x_{N}$, where N is the number of samples, the K-means algorithm finds k centroids $C=c_{1},\ldots ,c_{k}$ minimizing the mean distance between each data sample in x and their nearest centroids. The objective function is defined in Equation (7).

(7) $$E\left(C\right)=\frac{1}{N}\sum_{i=1}^{N}\left|\right|x_{i}-c\left(a_{i}\right){\left|\right|}^{2}$$

where *E* is the energy, and $a_{i}$ is the minimum distance between samples and centroids, i.e., $a_{i}=\arg\min_{j\in 1,\ldots ,k}\left|\right|x_{i}-c_{j}\left|\right|$.

There are many variations of K-means [49]; in our study, we employed the traditional Lloyd’s algorithm [44]. We did not perform comparisons with other clustering algorithms because our scope is centered on features and the information they extract. Moreover, previous work has shown that K-means is accurate and computationally efficient compared to others [47,49]. Additionally, several authors have succeeded in combining K-means clustering with Deep Neural Networks [48], suggesting it is a suitable choice for our purposes of exploring feature distributions in the data.

### 3.7. Performance Metrics

The following metrics have been employed to assess the performance of classification models in the supervised pathway:

- Accuracy: provides a global assessment of the model’s correctness by quantifying the ratio of correctly predicted instances to the total number of instances. Accuracy is defined in Equation (8).
  (8) $$\mathrm{Accuracy}=\frac{\mathrm{Number}\;\mathrm{of}\;\mathrm{Correct}\;\mathrm{Predictions}}{\mathrm{Total}\;\mathrm{Number}\;\mathrm{of}\;\mathrm{Instances}}$$
- Recall: also known as sensitivity, or the true positive rate, recall measures the model’s capability to accurately identify positive instances from the entire pool of actual positive instances, as depicted in Equation (9).
  (9) $$\mathrm{Recall}=\frac{\mathrm{True}\;\mathrm{Positives}}{\mathrm{True}\;\mathrm{Positives}\;+\;\mathrm{False}\;\mathrm{Negatives}}$$
- F1-score: the F1-score is obtained as the harmonic mean of precision and recall, offering a balanced measure that considers both false positives and false negatives, as described in Equation (10).
  (10) $$F1-\mathrm{score}=\frac{2\times \mathrm{Precision}\times \mathrm{Recall}}{\mathrm{Precision}\;+\;\mathrm{Recall}}$$

The following metrics have been employed to assess the quality of clustering models in the unsupervised pathway:

- Silhouette Coefficient: provides a measure of cluster cohesion and separation, as described in Equation (11). Cohesion is assessed based on the similarity of data instances within a single cluster, while separation is determined by the dissimilarity between instances from different clusters.
  (11) $$\mathrm{silhouette}\left(i\right)=\frac{b(i)-a(i)}{\max\{a(i),b(i)\}}$$
  where a(i) is the average distance from the *i*-th data point to other data points in the same cluster (cohesion) and b(i) is the smallest average distance from the *i*-th data point to data points in a different cluster, minimized over clusters (separation).
  The silhouette score for the entire dataset is the average of the silhouette score for each instance. The overall silhouette score can be calculated as in Equation (12).
  (12) $$\mathrm{silhouette}\_\mathrm{average}=\frac{\sum_{i=1}^{N}\mathrm{silhouette}\left(i\right)}{N}$$
- Calinski–Harabasz (CH) index: measures the ratio of between-cluster variance to within-cluster variance. It helps in assessing how well-separated the clusters are from each other. This index is calculated using Equation (13).
  (13) $$CHI=\frac{\mathrm{Tr}(B_{k})}{\mathrm{Tr}(W_{k})}\times \frac{N-k}{k-1}$$
  where $B_{k}$ is the between-cluster scatter matrix, $W_{k}$ is the within-cluster scatter matrix, *N* is the total number of data points, and *k* is the number of clusters.
- Davies–Bouldin (DB) index: computes the average similarity between each cluster and its most similar cluster. It provides insights into the compactness and separability of the clusters. The DB index is computed as in Equation (14).
  (14) $$DB=\frac{1}{n}\sum_{i=1}^{n}\max_{j\neq i}\left(\frac{\sigma_{i}+\sigma_{j}}{d(c_{i},c_{j})}\right)$$
  where *n* is the number of clusters, $\sigma_{i}$ is the average distance from the centroid of cluster *i* to the points in cluster *i*, $c_{i}$ is the centroid of cluster *i*, and $d(c_{i},c_{j})$ is the distance between centroids $c_{i}$ and $c_{j}$.

## 4. Experiments

We conducted experiments on the spectrograms generated from the Jaguas dataset processed as detailed in Section 3.1. We also considered metadata such as the timestamp of each recording, the recorder location, and the recording site cover type (forest or non-forest).

### 4.1. Autoencoder Architecture and Training

**Figure 3:**
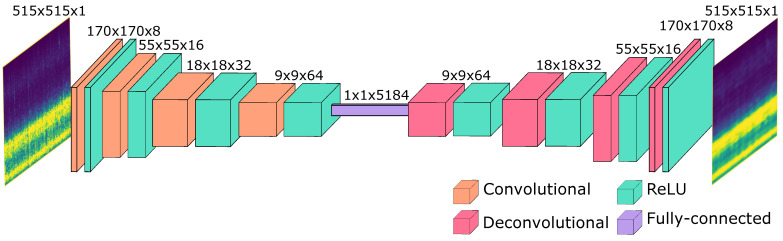
Proposed Autoencoder Neural Network. The embedded space is reduced to 5184 by applying a flattening to the residual image after the convolutional layers.

We allocated 98% of the dataset for training a vanilla autoencoder [35] with the architecture outlined in Figure 3. To assess the model’s performance and generalization capability, we reserved the remaining 2% of the dataset for testing purposes. During each epoch of training, this subset was utilized to evaluate the Mean Squared Error (MSE) as a test error metric. Additionally, spectrograms from this test subset were passed through the autoencoder to obtain reconstructions, enabling a visual comparison between the original and reconstructed spectrograms. This approach allowed us to monitor the autoencoder’s performance on unseen data and verify its capability to reconstruct spectrograms from the test set.

The encoding section of the autoencoder network comprises four convolutional layers with Rectified Linear Unit (ReLU) activation functions interspersed between them. Similarly, the decoding section follows the same structure, employing four deconvolutional layers with ReLU activation functions and a sigmoidal activation function for the final layer. The embedding space was derived using the encoding network by applying a flattening operation over the final layer. As a result, the dimension of the output was determined by the number of channels and the residual image after the convolutional layers, resulting in an embedding space of 5184 features. This outcome was achieved because the output channels were fixed to 64, and the residual image dimension was 9×9 (64×9×9=5184).

### 4.2. Supervised Learning Approach

We executed the supervised data processing pathway to compare the effectiveness of autoencoder features with more standard approaches in soundscape ecology. Considering that the dataset lacked information about biodiversity, species richness, or the presence of specific sound types, we relied on labels indicating the landscape type of the recording site, as either a forest or non-forest, provided by researchers involved in the sampling and analysis effort. We, thus, ran the RF classifier on the three input feature spaces:

- The autoencoder features, extracted from our trained autoencoder architecture.
- Feature vectors comprising sixty distinct acoustic indices computed using the scikit-maad Python module [50].
- The VGGish feature embedding, obtained from a pre-trained Convolutional Neural Network (17 layers) inspired by the VGG networks typically used for sound classification.

For the classification experiments, the dataset was divided into 80% of the samples for training and 20% for testing. We maintained uniform parameters and distributions across all classification models to ensure a direct comparison of results. In the case of the RF classifier, we set the maximum depth to 16 and the random state to 0. We applied dimensionality reduction to reduce the size of the feature space and use a feature space of the same size across all methods. The RF algorithm was then trained to predict landscape types using the reduced feature spaces obtained from each method.

Model performance was evaluated in terms of accuracy, recall, and F1-Score metrics, as described in Section 3.7. These metrics provide a comprehensive assessment of the performance of our classification models that take class imbalance into account. The analysis offers insights into the effectiveness of the three feature extraction techniques in predicting landscape types from the acoustic data.

### 4.3. Unsupervised Learning Approach

In the non-supervised data processing pathway, we employed the embedded autoencoder features to explore potential relationships between the temporal occurrences of acoustic events and the clusters identified with the K-means clustering algorithm. Furthermore, we employed PCA, t-SNE, and UMAP to generate two-dimensional projections of the features for visualization purposes. The visualizations are enriched with metadata concerning the recording hour and location in order to highlight potential temporal and spatial patterns. This allowed us to gain insight into the temporal and spatial distribution of acoustic events. By adopting this comprehensive approach, we were able to uncover valuable patterns and associations within the acoustic data, thereby contributing to a deeper understanding of the underlying dynamics of the soundscapes under investigation.

We evaluated the clustering quality using three commonly employed metrics: the Silhouette Coefficient (SLT), the Calinski–Harabasz (CH) index, and the Davies–Bouldin (DB) index. These metrics were applied while varying the number of clusters K from 3 to 35, allowing us to analyze the clustering performance across different cluster numbers.

## 5. Results

### 5.1. Autoencoder Training

**Figure 4:**
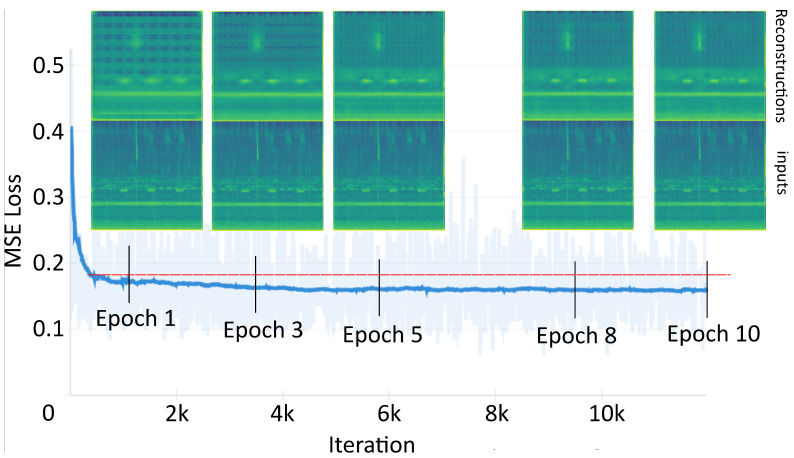
Autoencoder Learning Rate Curve: The similarity between reconstructions (**upper figures**) and original inputs (**lower figures**) was evaluated visually. Additionally, the difference between inputs and reconstructions was quantified using the Mean Squared Error (MSE) across ten epochs.

We conducted a thorough evaluation of the vanilla Autoencoder Neural Network, analyzing its learning rate curve over ten epochs. The Mean Squared Error (MSE) between the input and reconstructed spectrograms was computed, providing insights into the network’s performance. Additionally, we visually compared the reconstructions generated by the autoencoder with the corresponding input data samples. The autoencoder’s learning curve exhibited a rapid convergence, reaching the inflection point or “elbow” in less than one epoch (approximately 500 iterations) (Figure 4), indicating its efficiency. Following the inflection point, the mean error stabilized around 0.16, with a slight decrease observed in subsequent epochs (again, see Figure 4). Despite small fluctuations observed in the MSE across successive epochs, discernible improvements in the reconstructions were notable. Specifically, there was an accentuation of temporal patterns and an enhancement of background delineation. Accurate reconstructions of soundtype patterns, particularly in low and middle frequencies, were observed. However, challenges were encountered with higher frequencies beyond 8 kHz, where weak sounds became imperceptible in the reconstructions. Soundtypes with broad spectral ranges required multiple epochs to adjust to the original pattern. On the other hand, concerning background noise, the network initially depicted remarkable repeated patterns, gradually diminishing with each epoch. This Deep Neural Network was employed to extract a feature embedding, which served as an input for both the supervised and unsupervised data analysis pathways.

### 5.2. Feature Projections

**Figure 5:**
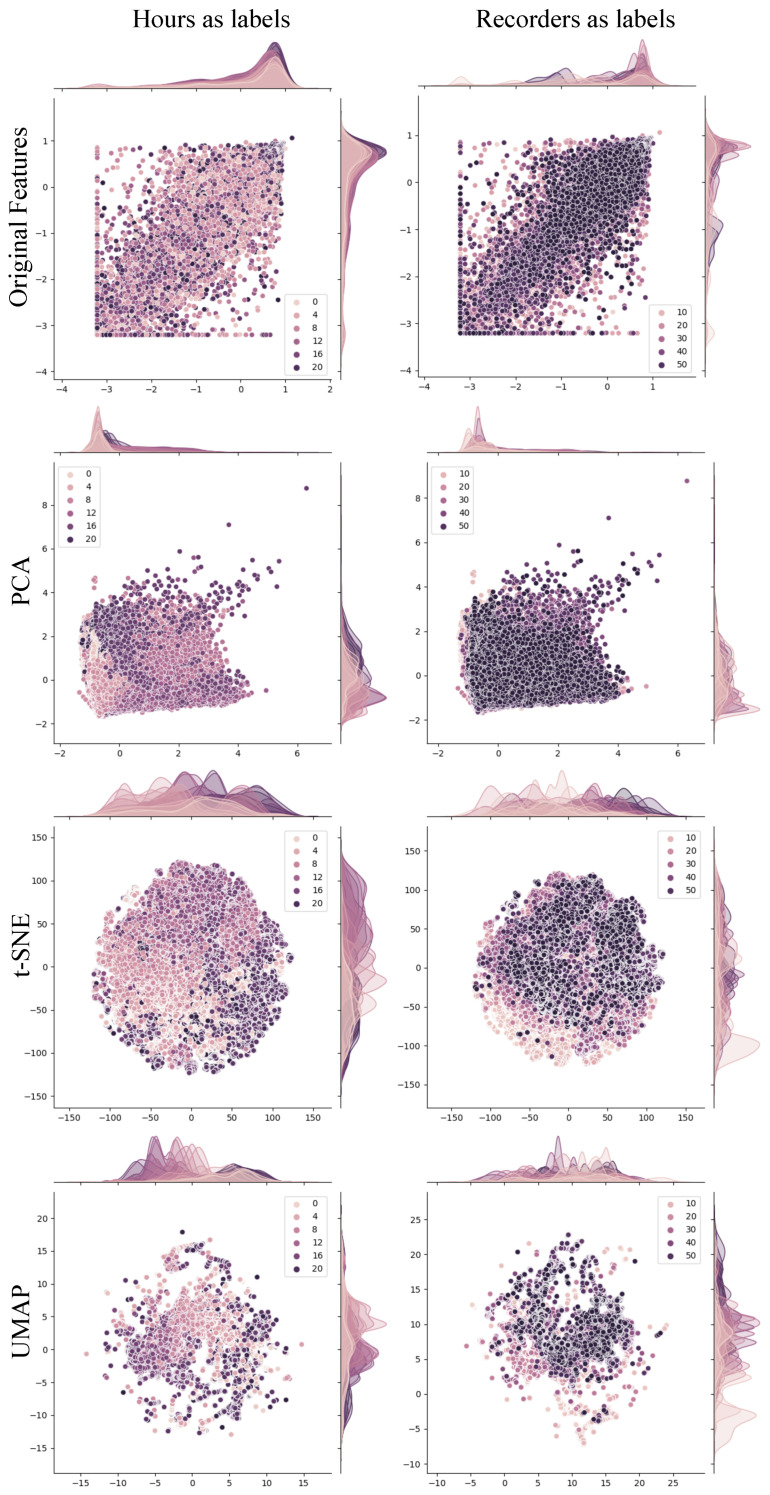
The projections of the feature space obtained with PCA, t-SNE, and UMAP are displayed in the rows. The points in the plots represent the audio segments, each distinct color shade maps a specific hour or location label. This setup favors a comparative analysis of data distributions across different dimensions and labeling schemes.

We generated reduced embeddings of the original autoencoder’s feature space using various dimensionality reduction techniques. Initially, we employed the traditional Principal Component Analysis (PCA) method to identify and organize components based on decreasing variance. This approach effectively determined a minimal number of components that accounted for over 90% of the data variance, facilitating feature space reduction while preserving performance and information content. Although 30 components captured 90% of the variance, we opted for 60 components to align with the dimensionality of the feature vector of acoustic indices. In addition to PCA, we utilized T-SNE and UMAP for dimensionality reduction, considering them state-of-the-art methods. We preserved the same number of components (60) to maintain consistency across feature vectors. Consequently, feature vectors of size 60 were input to the classification and clustering tasks. For visualization purposes, only two dimensions per method were used to showcase data distribution, as depicted in Figure 5. By examining these visualizations, one can discern patterns and relationships within the data based on the recording hour or location labels, providing insights into the temporal and spatial distributions of acoustic events across the dataset.

In Figure 5, we compare distributions using the recording time and recorder location as labels for each data point. In terms of the temporal aspect, PCA effectively distinguishes data points across different hours. However, more favorable outcomes were observed in the views obtained with UMAP and t-SNE, which demonstrated superior capability to separate hours into distinct distributions. On the other hand, discerning a clear pattern in the distribution of recording locations is not straightforward. Nevertheless, UMAP and t-SNE again exhibited superior separation for certain segregated points. Moreover, the absence of discernible segregation among points corresponding to various recording locations is regarded as a favorable outcome, as we did not expect to discover notorious patterns that might be introduced from the bias of the recorders. Therefore, we consider that spatial patterns deserve further investigations, given the lack of distinct separations, indicating that a more in-depth exploration of potential spatial correlations is required.

### 5.3. Classification of Landscape Type Using Supervised Learning

**Figure 6:**
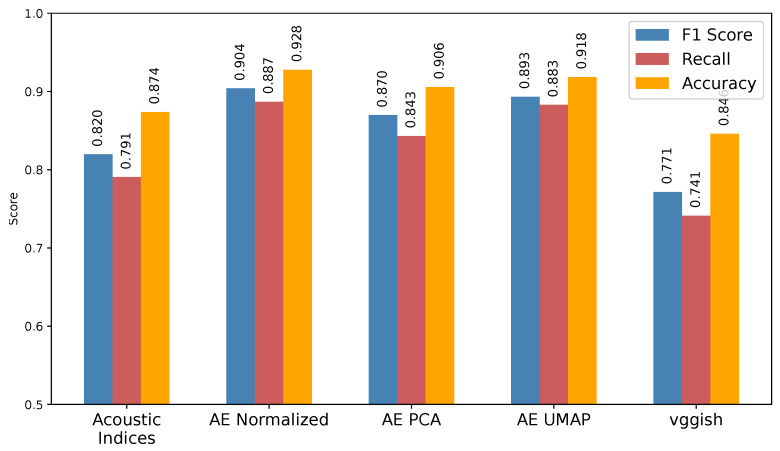
Results of classification with Random Forest using autoencoder features (before and after dimensionality reduction) as compared with a vector of acoustic indices and VGGish features.

We trained a Random Forest classifier using the data point labels of “forest” or “non-forest”, considering the reduced feature spaces relative to the three feature extraction methodologies. The forest and non-forest classification was based on a land cover map derived from satellite images. Forest cover exclusively included forested areas, while non-forest cover comprised all other land cover types (such as grasslands and secondary vegetation) [51]. Our objective in this experiment was to compare the performance of the features obtained with the proposed autoencoder with methods recognized as effective in the literature, such as acoustic indices and the VGGish architecture. Additionally, we aimed to enrich our analysis by incorporating contextual information. Specifically, we were interested in leveraging satellite-derived labels and biological content extracted from the audio of the sites. This was motivated by the understanding that various factors, including degradation, landscape transformation, and other conditions, can interfere with acoustic patterns. Figure 6 presents the results pertaining to the original autoencoder embedding and after applying dimensionality reduction to 60 components, along with the acoustic indices and VGGish features. These results offer insights into the effectiveness of different feature extraction methodologies in distinguishing between forest and non-forest environments.

The normalized autoencoder features exhibited the best classification metrics, with an F1 score of 90.4%, recall of 88.7%, and accuracy of 92.8%. Additionally, its projections, particularly with UMAP and 60 components, yielded comparable results, indicating that reducing the original feature space from 5184 to 60 components is computationally efficient while preserving relevant information. While PCA-based projections showed improved results compared to the baseline methodologies of acoustic indices and VGGish, there was a notable discrepancy compared to the original features and UMAP. This suggests that UMAP has superior capacity to compress relevant information in certain contexts. However, it is worth noting that results may vary depending on the specific task and landscape attribute being assessed. Conversely, the feature spaces computed with the baseline methodologies exhibited comparatively inferior performance and demonstrated limitations despite their frequent use. In the case of VGGish, although it computes spectrograms approximately every minute, compared to our solution which computes spectrograms every twelve seconds, there is a significant difference in all the classification metrics, indicating that spectrograms extracted from longer time series than those reported in the state of the art can reveal discriminant patterns.

### 5.4. Unsupervised Learning Results

To explore the potential of unsupervised methods in understanding landscape heterogeneity patterns without relying on species-specific analysis, we investigated temporal and sound type relationships within the clusters obtained with K-means, following the methodology outlined in [52]. We systematically generated clusters with K ranging from three to thirty. For each clustering iteration, we computed both individual and average Silhouette scores (SLT), as well as the Davies–Bouldin and Calinski–Harabasz indices, serving as metrics of cluster quality.

Table 3 shows the results for models obtained with varying values of K and four input feature spaces (the autoencoder features and the three-dimensional reduced spaces obtained with PCA, t-SNE and UMAP). One observes that the best scores in all metrics were obtained with three clusters in most cases, and the scores tended to decrease as the number of clusters increased. However, there are a few exceptions, e.g., the UMAP features yielded better DB index scores with 35 clusters, whereas the t-SNE features yielded the best DB index with 7 clusters and a better CH index with 30 clusters. Notably, the UMAP features yielded better cluster quality metrics, indicating they consistently excelled in creating well-defined, compact, and segregated clusters, yielding an SLT of 0.41, DB index of 0.85, and CH index of 86,427.27, as detailed in Table 3. Moreover, when computing the metrics’ means and standard deviations, UMAP showed the best mean values for SLT and CH and the best deviation value for DB; in contrast, t-SNE had the best values of DB mean and Silhouette and CH deviations. Nevertheless, both t-SNE and PCA contributed to score improvements, with PCA showing a slight enhancement, and t-SNE achieved the highest DB index using seven clusters as mentioned before, indicating that although UMAP and t-SNE are similar theoretically, spaces projected by these methods are well differentiable. On the other hand, contrasting the number of clusters among projection methods, we identified that when clusters increased, several patterns in alternating bands of frequency were accentuated, indicating that clusters could be considered similar based on the quantitative metrics, but there were variations in the biological content associated with animal vocalizations, i.e., for unsupervised studies, the metrics should be aligned to the biological content of recordings due to clusters with lower scores being able to capture relevant information and patterns which are sensitive to being masked by other frequency ranges.

**Figure 7:**
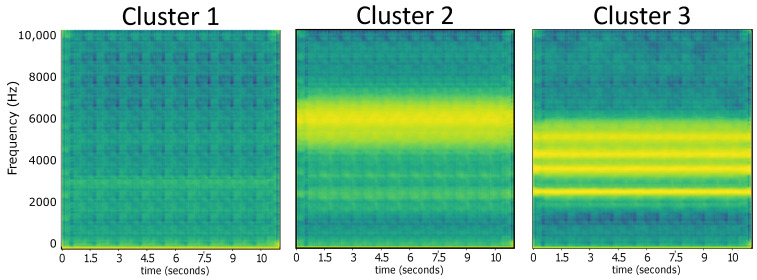
Centroid spectrograms computed by passing the feature vector of each cluster centroid to the decoder part of the autoencoder.

We have confirmed our hypothesis regarding the effectiveness of deep features in revealing macro patterns within the soundscape. We computed centroid spectrograms by passing the centroid feature vector in each cluster to the decoder part of the autoencoder. In this manner, we reconstructed spectrograms associated with inputs generated by the K-means clustering algorithm. This process allowed us to visualize the representative spectrograms corresponding to each cluster, providing insights into the characteristic acoustic patterns captured by the clusters (refer to Figure 7). This part of our methodology is a novel way to represent clusters’ information for soundscape applications due to the fact that, in general, cluster prototypes are ignored in clustering procedures, despite containing valuable information about the composition of the clusters.

Specifically, the distinct centroid spectrograms (refer to Figure 7) effectively delineate noticeable changes in frequency bands, primarily reflecting distinct occupied bands. Our analysis revealed that cluster one mostly represented a quiet soundscape. Upon further investigating the cluster contents, we found mainly light rainfall, some insect sounds, and natural sounds like river sounds for cluster one. Given the frequent occurrence of cicadas and rainfall in most recordings and the study area, this result was expected. Cluster two also featured recordings of insects, albeit with additional biophonies such as anuran vocalizations represented in lower frequency ranges around 2 KHz, as it can be seen in Figure 7. Cluster three captured a medium-high spectral band, predominantly around 4 kHz, showcasing anuran and bird vocalizations; also, we can specify that these patterns correspond to animal vocalizations because there are accentuated intensities for thin frequency bands (2 Khz to 3 Khz, 3.5 Khz to 4 Khz, and 5 Khz to 6 Khz). These frequency ranges are commonly utilized by animals as part of their acoustic niche and are relevant for studying bioacoustic richness [53].

**Figure 8:**
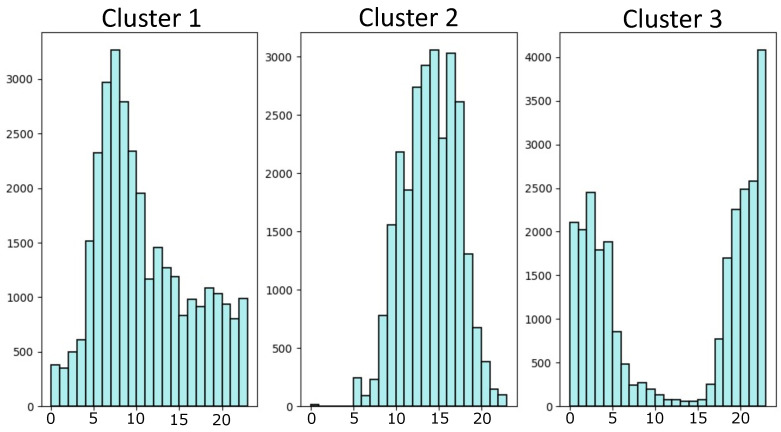
Histograms of audio membership in each cluster using K-Means and UMAP projections at different times of day.

Finally, we computed histograms for the three clusters discussed above to examine temporal trends within the groups, as depicted in Figure 8. This analysis unveiled distinct temporal distributions among the clusters. For instance, we discerned two primary time intervals in the three clusters. The most pronounced temporal interval spanned clusters three, encompassing a nocturnal time-frame from 6 p.m. to 5 a.m. Given Colombia’s proximity to the equator, the sunrise and sunset hours remain nearly the same all year-round, typically around 6 a.m. and 6 p.m., respectively. A diurnal time-frame was prevalent in the remaining clusters. However, it is noteworthy that cluster two exhibited broader temporal ranges. Most recordings in cluster two primarily spanned 10 a.m. to 6 p.m., while in cluster one, recordings between 4 a.m. and 11 a.m. prevailed. The latter indicates the presence of acoustic landscapes characterized by silence, indicating a minimal detection of biophonies, geophonies, and anthropophonies.

## 6. Discussion and Future Work

We introduce an unsupervised learning framework based on an autoencoder architecture to extract representative features from soundscapes of a Colombian region, in association with the K-means clustering algorithm to reveal patterns present in acoustic recordings. Following ten epochs, we assessed the accuracy of the representations using Mean Squared Error (MSE) and the learning rate curve of the Deep Neural Network, and we conducted a visual inspection by comparing spectrogram inputs and outputs. During evaluation, we identified challenges in assessing the reconstructions despite achieving low MSE values. The absence of a defined baseline or cut-off point made it difficult to establish a clear threshold to assess reconstruction accuracy. We addressed this issue by conducting supervised learning experiment using landscape labels to discriminate between forest and non-forest areas. This allowed us to compare the performance of autoencoder features with baseline methodologies such as acoustic indices and VGGish. The classification results revealed that autoencoder features outperformed several metrics, especially after reducing the feature space with dimensional reduction methods (PCA, t-SNE adn UMAP). Despite UMAP not yielding the highest classification scores, it significantly reduced the embedded space while maintaining performance close to that obtained using the full feature space.

For the unsupervised learning experimentation, because UMAP allows us to project back features to the original space, we could create centroid spectrograms, which are spectrograms made from the reduced information of the reduced space, giving a better interpretability to the clusters and guiding along with conventional metrics such as Silhouette for the selection of a correct number of clusters. Additionally, employing metadata of the dataset was possible to establish temporal dynamics which are associated with acoustic biodiversity represented by the occupied range of frequencies.

In the unsupervised learning experimentation, as the autoencoder enables the projection of features back to the original space, we could generate cluster centroid spectrograms from the reduced information of the feature space. These spectrograms enhanced cluster interpretability, and in conjunction with conventional metrics, they can aid in finding an appropriate number of clusters. Additionally, the metadata of the dataset enabled us to establish temporal dynamics associated with acoustic biodiversity in the clusters, represented by the occupied range of frequencies.

We are confident that our proposal will expand the research horizons of non-species-specific soundscape studies, further exploring the multifaceted dimensions of biocomplexity. Nevertheless, ongoing work persists, especially in unsupervised studies within soundscape and ecoacoustics. Establishing a precise distribution of the data remains challenging, given the presence of not only explicit and observable patterns but also intricate dynamics within ecosystems. Deep learning algorithms hold promise in capturing these complex dynamics; however, challenges persist in interpreting and understanding ecological behaviors. Despite these obstacles, our efforts contribute to advancing the field by addressing these complexities and striving for deeper insights into the dynamics of soundscapes and ecoacoustic environments.

## 7. Conclusions

Despite the significant advances and remarkable progress in leveraging machine learning for ecoacoustic analysis, several challenges persist regarding conservation and monitoring strategies. A primary obstacle is the limited availability of labeled data essential for training machine learning algorithms, hindering the accurate recognition of vocalizations at the species level for animals such as birds and insects. Moreover, the dynamic and intricate nature of the acoustic landscape demands algorithms capable of handling vast amounts of data in real time and scaling to manage the environment’s complexity effectively. Thus, ecoacoustics, on a large scale, is imperative to glean environmental attributes and changes. Unsupervised learning emerges as a practical solution for analyzing and exploring soundscapes capable of addressing some of these challenges. Unsupervised learning offers several advantages, including independence from labels, versatility in examining multiple sound sources present in recordings, and the automatic exploration of relationships among acoustic patterns. However, it is crucial to note that unsupervised learning relies on informative features to perform clustering effectively. Efforts to develop robust feature representations remain integral to advancing unsupervised learning methods in ecoacoustic analysis.

Our findings pave the way for further explorations in soundscape ecology, advocating for the integration of unsupervised learning approaches to gain a deeper understanding of landscape heterogeneity. The success of autoencoder-based features in classification tasks, alongside the identification of meaningful patterns in unsupervised clustering, highlights their potential as valuable tools in soundscape research. Moreover, our research underscores the importance of selecting appropriate dimensionality reduction techniques for unsupervised learning in soundscape studies. UMAP emerges as a robust method, demonstrating its ability to reveal meaningful patterns and heterogeneity within acoustic landscapes. Moving forward, future research endeavors should prioritize the refinement of spatial analysis techniques, the exploration of alternative unsupervised learning methodologies, and a comprehensive comparison of ecological and biological information across various dimensionality reduction methods tailored to the specified number of clusters. These initiatives will further enhance our understanding of acoustic environments and their ecological significance.

## Figures and Tables

**Table 1:** Review of related work: Our selection criteria focused on articles that utilize autoencoders for representing soundscape data, perform clustering analysis on spectrogram data, and introduce novel methods for enhancing clustering performance and integrating features into clustering for embedded representations.

Approach	Methods	Dataset	Reference	Year
Unsupervised	UEC, GMMs, NMI, K-means	IRIS, Spiral, CIFAR10, ATOM, EngyTime, USPS, MNIST, Reuters 10K	[24]	2024
Supervised	VGG, UMAP, HDBSCAN	8 datasets of timestamped and type labeled vocalizations of birds and marine mammals	[21]	2023
Supervised	Q Wavelets, 1D-LBP, KNN	New anuran dataset with 1536 sounds of 26 species	[22]	2023
Supervised and Unsupervised	Variational Autoencoders, UMAP, Binomial classification	Datasets from Equador and United Kingdom about habitat degradation	[15]	2023
Unsupervised	GMMs, Uncertainty Fréchet	36 Synthetic and 5 real datasets (including a PAM dataset)	[23]	2023
Unsupervised	Acoustic indices, Autoencoders, Hierarchical clustering	SERF Dataset	[19]	2021
Unsupervised	Acoustic indices, Image descriptors, Autoencoders, PCA, t-SNE, LAMP	More than 4000 files from terrestrial and marine ecosystems from Costa Rica and Brazil	[20]	2021

**Table 2:** Data acquisition and sampling parameters: Rec refers to the recorders, NSS to the number of sites studied, RD to the recording duration, SR to the sample rate, SS to the sub-sampling rate, RP to the periodicity of recording, MU to the memory used to store the recordings, AL to the cover type labels available across sites.

	Rec	NSS	RD (S)	SR (KHz)	SS (KHz)	dB Gain	RP	MU (GB)	AL	
	SM4	31	60	44.1	22.05	16	1 min every	212.4	Forest	Non-forest
							15 min		14,637	5431
Total									20,068	

**Table 3:** Quality metrics (Silhouette coefficient (SLT), Davies–Bouldin index (DB), and Calinski–Harabasz index (CH)) of cluster models obtained with K-means over the autoencoder features and UMAP, t-SNE, and PCA projections, for varying values of K. The best values for each configuration are shown in bold, and the best global values are shown in blue. SLT values are in the range of [−1,1], where higher values indicate better cohesion and separation. Lower values of DB are better, indicating dense and well-segregated clusters, whereas higher values of CH index are better.

NC	Full Embedded Space			UMAP			t-SNE			PCA		
	SLT	DB	CH	SLT	DB	CH	SLT	DB	CH	SLT	DB	CH
3	**0.2131**	**1.7692**	**29,185**	**0.4123**	0.8589	**86,427**	**0.3783**	0.8565	66,293	**0.2493**	**1.5490**	**37,770**
5	0.1368	2.0666	20,519	0.3872	0.8167	85,305	0.3411	0.9031	70,358	0.1773	1.7393	27,896
7	0.1304	1.9938	16,484	0.3503	0.8709	81,715	0.3646	**0.7740**	74,417	0.1755	1.6660	23,248
10	0.128	2.0494	12,910	0.3420	0.8665	78,940	0.3431	0.8495	72,635	0.1753	1.6801	18,927
15	0.1032	2.0458	9707	0.3373	0.8312	78,438	0.3412	0.8137	73,335	0.1537	1.6943	14,755
20	0.1000	2.1764	7766	0.3294	0.8284	77,257	0.3406	0.7807	73,952	0.1483	1.7404	12,251
25	0.0839	2.2417	6556	0.3262	0.8287	76,505	0.3406	0.7826	74,813	0.1428	1.7605	10,572
30	0.0826	2.259	5719	0.3254	0.8194	76,092	0.3425	0.7883	**74,835**	0.1321	1.7655	9361
35	0.0786	2.2819	5092	0.3309	**0.8145**	76,371	0.3403	0.7956	74,798	0.1304	1.7801	8490
**Mean**	0.1174	2.0982	12,660	**0.3490**	0.8372	**79,672**	0.3480	**0.8160**	72,826	0.1649	1.7083	18,141
**STD**	0.03	0.15	7637	0.02	**0.02**	3699	**0.01**	0.04	**2682**	0.03	0.06	9314

## Data Availability

Dataset and software codes are available under request.

## References

[1] Quinn C.A., Burns P., Gill G., Baligar S., Snyder R.L., Salas L., Goetz S.J., Clark M.L. (2022). Soundscape classification with convolutional neural networks reveals temporal and geographic patterns in ecoacoustic data. Ecol. Indic..

[2] Gan H., Zhang J., Towsey M., Truskinger A., Stark D., van Rensburg B.J., Li Y., Roe P. (2020). Data selection in frog chorusing recognition with acoustic indices. Ecol. Inform..

[3] Siddagangaiah S., Chen C.F., Hu W.C., Akamatsu T., McElligott M., Lammers M.O., Pieretti N. (2020). Automatic detection of dolphin whistles and clicks based on entropy approach. Ecol. Indic..

[4] Fink D., Auer T., Johnston A., Ruiz-Gutierrez V., Hochachka W.M., Kelling S. (2020). Modeling avian full annual cycle distribution and population trends with citizen science data. Ecol. Appl..

[5] Mitchell S.L., Bicknell J.E., Edwards D.P., Deere N.J., Bernard H., Davies Z.G., Struebig M.J. (2020). Spatial replication and habitat context matters for assessments of tropical biodiversity using acoustic indices. Ecol. Indic..

[6] Lahoz-Monfort J.J., Magrath M.J. (2021). A Comprehensive Overview of Technologies for Species and Habitat Monitoring and Conservation. BioScience.

[7] Gibb R., Browning E., Glover-Kapfer P., Jones K.E. (2019). Emerging opportunities and challenges for passive acoustics in ecological assessment and monitoring. Methods Ecol. Evol..

[8] Irfan M., Mushtaq Z., Khan N.A., Althobiani F., Mursal S.N.F., Rahman S., Magzoub M.A., Latif M.A., Yousufzai I.K. (2023). Improving Bearing Fault Identification by Using Novel Hybrid Involution-Convolution Feature Extraction With Adversarial Noise Injection in Conditional GANs. IEEE Access.

[9] Shinde P.P., Shah S. A Review of Machine Learning and Deep Learning Applications. Proceedings of the 2018 4th International Conference on Computing, Communication Control and Automation, ICCUBEA 2018.

[10] Keen S.C., Odom K.J., Webster M.S., Kohn G.M., Wright T.F., Araya-Salas M. (2021). A machine learning approach for classifying and quantifying acoustic diversity. Methods Ecol. Evol..

[11] Brodie S., Allen-Ankins S., Towsey M., Roe P., Schwarzkopf L. (2020). Automated species identification of frog choruses in environmental recordings using acoustic indices. Ecol. Indic..

[12] Lauha P., Panu S., Petteri L., Lisa G., Tobias R., Sebastian S., Ovaskainen O. (2022). Domain-specific neural networks improve automated bird sound recognition already with small amount of local data. Methods Ecol. Evol..

[13] Dufourq E., Batist C., Foquet R., Durbach I. (2022). Passive acoustic monitoring of animal populations with transfer learning. Ecol. Inform..

[14] Zhang C., Zhan H., Hao Z., Gao X. (2023). Classification of Complicated Urban Forest Acoustic Scenes with Deep Learning Models. Forests.

[15] Gibb K.A., Eldridge A. (2024). Towards Interpretable Learned Representations for Ecoacoustics Using Variational Auto-Encoding. Ecol. Inform..

[16] Hilasaca L.H., Ribeiro M.C., Minghim R. (2021). Visual active learning for labeling: A case for soundscape ecology data. Information.

[17] Sun Y.J., Yen S.C., Lin T.H. (2022). soundscape IR: A source separation toolbox for exploring acoustic diversity in soundscapes. Methods Ecol. Evol..

[18] Ulloa J.S., Aubin T., Llusia D., Bouveyron C., Sueur J. (2018). Estimating animal acoustic diversity in tropical environments using unsupervised multiresolution analysis. Ecol. Indic..

[19] Rowe B., Eichinski P., Zhang J., Roe P. (2021). Acoustic auto-encoders for biodiversity assessment. Ecol. Inform..

[20] Dias F.F., Pedrini H., Minghim R. (2021). Soundscape segregation based on visual analysis and discriminating features. Ecol. Inform..

[21] Best P., Paris S., Glotin H., Marxer R. (2023). Deep audio embeddings for vocalisation clustering. PLoS ONE.

[22] Akbal E., Barua P.D., Dogan S., Tuncer T., Acharya U.R. (2023). Explainable automated anuran sound classification using improved one-dimensional local binary pattern and Tunable Q Wavelet Transform techniques. Expert Syst. Appl..

[23] Rendon N., Giraldo J.H., Bouwmans T., Rodríguez-Buritica S., Ramirez E., Isaza C. (2023). Uncertainty clustering internal validity assessment using Fréchet distance for unsupervised learning. Eng. Appl. Artif. Intell..

[24] Allaoui M., Kherfi M.L., Cheriet A., Bouchachia A. (2024). Unified embedding and clustering. Expert Syst. Appl..

[25] Nieto-Mora D.A., Rodríguez-Buritica S., Rodríguez-Marín P., Martínez-Vargaz J.D., Isaza-Narváez C. (2023). Systematic review of machine learning methods applied to ecoacoustics and soundscape monitoring. Heliyon.

[26] Hershey S., Chaudhuri S., Ellis D.P., Gemmeke J.F., Jansen A., Moore R.C., Plakal M., Platt D., Saurous R.A., Seybold B. CNN architectures for large-scale audio classification. Proceedings of the ICASSP, IEEE International Conference on Acoustics, Speech and Signal Processing.

[27] Cano Rojas E., Sanchez Giraldo C., Bedoya C., Daza J.M. (2022). Hábitat y espectro acústico como factores determinantes de la ocupación de anuros neotropicales. Biota Colomb..

[28] Sánchez-Giraldo C., Correa Ayram C., Daza J.M. (2021). Environmental sound as a mirror of landscape ecological integrity in monitoring programs. Perspect. Ecol. Conserv..

[29] Bedoya C., Isaza C., Daza J.M., López J.D. (2017). Automatic identification of rainfall in acoustic recordings. Ecol. Indic..

[30] Pahuja R., Kumar A. (2021). Sound-spectrogram based automatic bird species recognition using MLP classifier. Appl. Acoust..

[31] Zhong M., LeBien J., Campos-Cerqueira M., Dodhia R., Lavista Ferres J., Velev J.P., Aide T.M. (2020). Multispecies bioacoustic classification using transfer learning of deep convolutional neural networks with pseudo-labeling. Appl. Acoust..

[32] Steinfath E., Palacios-Muñoz A., Rottschäfer J.R., Yuezak D., Clemens J. (2021). Fast and accurate annotation of acoustic signals with deep neural networks. eLife.

[33] Mushtaq Z., Su S.F. (2020). Efficient classification of environmental sounds through multiple features aggregation and data enhancement techniques for spectrogram images. Symmetry.

[34] Ventura T.M., De Oliveira A.G., Ganchev T.D., De Figueiredo J.M., Jahn O., Marques M.I., Schuchmann K.L. (2015). Audio parameterization with robust frame selection for improved bird identification. Expert Syst. Appl..

[35] Hinton G., Salakhutdinov R.R. (2006). Reducing the Dimensionality of Data with Neural Networks. Int. Encycl. Educ..

[36] Tan W.G.Y., Xiao M., Wu Z. (2024). Robust reduced-order machine learning modeling of high-dimensional nonlinear processes using noisy data. Digit. Chem. Eng..

[37] Vincent P., Larochelle H. (Denoising AE) Extracting and Composing Robust Features with Denoising. Proceedings of the 25th International Conference on Machine Learning.

[38] Dong G., Liao G., Liu H., Kuang G. (2018). A Review of the Autoencoder and Its Variants: A Comparative Perspective from Target Recognition in Synthetic-Aperture Radar Images. IEEE Geosci. Remote Sens. Mag..

[39] Kammoun A., Ravier P., Buttelli O. (2024). Impact of PCA Pre-Normalization Methods on Ground Reaction Force Estimation Accuracy. Sensors.

[40] Siddique M.F., Ahmad Z., Ullah N., Kim J. (2023). A Hybrid Deep Learning Approach: Integrating Short-Time Fourier Transform and Continuous Wavelet Transform for Improved Pipeline Leak Detection. Sensors.

[41] Van der Maaten L., Geoffrey H. (2008). Visualizing Data using t-SNE Laurens. Ann. Oper. Res..

[42] McInnes L., Healy J., Melville J. (2018). UMAP: Uniform Manifold Approximation and Projection for Dimension Reduction. arXiv.

[43] Urrutia R., Espejo D., Evens N., Sühn T., Boese A., Hansen C., Fuentealba P., Illanes A., Poblete V. (2023). Clustering Methods for Vibro-Acoustic Sensing Features as a Potential Approach to Tissue Characterisation in Robot-Assisted Interventions. Sensors.

[44] Morissette L., Chartier S. (2013). The k-means clustering technique: General considerations and implementation in Mathematica. Tutor. Quant. Methods Psychol..

[45] Sinaga K.P., Yang M.S. (2020). Unsupervised K-means clustering algorithm. IEEE Access.

[46] Khan A., Hao J., Dong Z., Li J. (2023). Adaptive Deep Clustering Network for Retinal Blood Vessel and Foveal Avascular Zone Segmentation. Appl. Sci..

[47] Ikotun A.M., Ezugwu A.E., Abualigah L., Abuhaija B., Heming J. (2023). K-means clustering algorithms: A comprehensive review, variants analysis, and advances in the era of big data. Inf. Sci..

[48] Yang H., Wang J., Wang J. (2023). Efficient Detection of Forest Fire Smoke in UAV Aerial Imagery Based on an Improved Yolov5 Model and Transfer Learning. Remote Sens..

[49] Abu Abbas O. (2008). Comparisons Between Data Clustering Algorithms. Int. Arab. J. Inf. Technol..

[50] Ulloa J.S., Haupert S., Latorre J.F., Aubin T., Sueur J. (2021). scikit-maad: An open-source and modular toolbox for quantitative soundscape analysis in Python. Methods Ecol. Evol..

[51] Sánchez-Giraldo C., Bedoya C.L., Morán-Vásquez R.A., Isaza C.V., Daza J.M. (2020). Ecoacoustics in the rain: Understanding acoustic indices under the most common geophonic source in tropical rainforests. Remote Sens. Ecol. Conserv..

[52] Zhou H.B., Gao J.T. (2014). Automatic method for determining cluster number based on silhouette coefficient. Adv. Mater. Res..

[53] Sousa-Lima R.S., Ferreira L.M., Oliveira E.G., Lopes L.C., Brito M.R., Baumgarten J., Rodrigues F.H. (2018). What do insects, anurans, birds, and mammals have to say about soundscape indices in a tropical savanna. J. Ecoacoust..

